# The Association between Quality Improvement Initiatives in Dementia Care and Supportive Psychosocial Work Environments in Nursing Homes

**DOI:** 10.3390/healthcare6020044

**Published:** 2018-05-08

**Authors:** Miharu Nakanishi, Maki Tei-Tominaga

**Affiliations:** 1 Mental Health and Nursing Research Team, Tokyo Metropolitan Institute of Medical Science, 2-1-6 Kamikitazawa, Setagaya-ku, Tokyo 156-8506, Japan; 2 Faculty of Nursing, Setsunan University, 45-1 Nagaotoge-cho, Hirataka-shi, Osaka 573-0101, Japan; maki.tominaga@nrs.setsunan.ac.jp

**Keywords:** dementia, nursing home staff, professionalism, psychosocial work environment

## Abstract

*Background*: Quality improvement initiatives can help nursing homes strengthen psychosocial work environments. The aim of the present study was to examine the association between supportive psychosocial work environment, and professional and organizational characteristics regarding quality improvement initiatives in dementia care. *Methods*: A paper questionnaire survey was administered to a convenience sample of 365 professional caregivers in 12 special nursing homes in Japan. Psychosocial work environment was assessed using the Social Capital and Ethical Climate at the Workplace Scale to calculate a score of social capital in the workplace, ethical leadership, and exclusive workplace climate. Variables for quality improvement initiatives included type of home (unit-type or traditional), presence of additional benefit for dementia care, and professionalism in dementia care among caregivers evaluated using the Japanese version of the Sense of Competence in Dementia Care Staff Scale. *Results*: Elevated professionalism and unit-type home were significantly associated with high social capital, strong ethical leadership, and low exclusive workplace climate. The presence of dementia care benefit was not associated with any subscale of psychosocial work environment. *Conclusions*: Quality improvement initiatives to foster supportive psychosocial work environment should enhance professionalism in dementia care with unit-based team building of professional caregivers in special nursing homes.

## 1. Introduction

The importance of workforce strategy in nursing homes has increased. The number of people living with dementia has risen worldwide [1]. A large portion of nursing home residents have dementia and the majority of people with dementia have received end-of-life care at nursing homes [2,3,4]. However, several nursing homes face high turnover and shortage in care staff [5].

Psychosocial work environment plays an important role in workforce retention of nursing homes. Psychological work environment refers to attitudes and perceptions that can influence the performance of an organization’s members [6]. Supportive environments at workplaces modify the impact of workload on physical and mental health outcomes [7]. In addition to the positive components, negative environments where exclusive climate exists, or harassment and mobbing occur, can affect health and well-being [8] and intention to leave among members [9]. Nursing home staff tended to experience poor psychosocial workplace environments compared to nurses in other sectors [10]. Elucidating the professional and organizational characteristics relating to supportive psychosocial work environments might help nursing homes identify areas for intervention to improve workforce retention. Quality improvement initiatives can help special nursing homes communicate their mission to professional caregivers and strengthen psychosocial work environments. In Western nursing homes, good quality dementia care has been found to be closely related to a supportive psychosocial work environment [11,12,13].

Japan introduced a public long-term care insurance system in 2000 where a universal benefit schedule is applied to long-term care services. Special nursing homes are the only facilities available for permanent residence under public long-term residential care services. The majority of residents in special nursing homes had a certain level of cognitive impairment [14]. There has been a long waiting list for nursing home placement (295,237 applicants in March 2017) [15] compared with number of places available (530,280 in October 2016) [16] and a serious shortage of care staff due to high turnover in special nursing homes [17,18]. Only local governments and social welfare corporations (non-profit organization) are permitted to run special nursing homes. The public long-term care insurance program sets a fixed amount of benefits for basic residential care package per resident regardless of differences in insurers [19,20].

The Japanese government has introduced “unit-type” homes and additional benefit for dementia care aiming at quality improvement initiatives in special nursing homes. Traditional special nursing homes are based on the medical model and usually have shared bedrooms for residents. Unit-type homes have private rooms and residential units where frail older adults can spend time alone in their own rooms in a small, homelike environment. Each residential unit provides a common area, such as a dining room for interaction among residents, and has stable staff assignments [21]. Small-scale environments require more focus on individual needs and preferences of the resident and integrated way of working resulting in higher social support in the workplace compared to traditional environments based on the medical model and task-oriented way of working [22]. An additional benefit for dementia care is being certified by the national government to receive payment for the specialized dementia care package and treatment of responsive behavior in addition to the basic residential care benefit. Certification of the additional benefit for dementia care requires a facility to have direct care workers who have received specialized dementia care training. However, little is known about quality improvement initiatives in dementia care and psychosocial work environments among Japanese special nursing homes.

The aim of the present study was to examine the association between supportive psychosocial work environments and professional and organizational characteristics regarding quality improvement initiatives in dementia care in Japanese nursing homes. We hypothesized that the professional caregivers reported supportive psychosocial work environment when they worked in special nursing homes with quality improvement initiatives in dementia care.

## 2. Materials and Methods

### 2.1. Design

A cross-sectional study design was used to collect data on professional caregivers and organizations regarding quality improvement initiatives in Japanese nursing homes within a 4-month period between August and November 2017.

### 2.2. Setting

A total of 12 special nursing homes across 3 prefectures were invited to participate in this study. In the 12 special nursing homes, 4 (33.3%) acquired an additional benefit for dementia care. Nine (75.0%) facilities were unit-type homes. The location was selected based on the ratio of active job openings to applicants in long-term care in 2016 (Table 1). The 12 facilities that were recruited were part of the researchers’ knowledge network pertaining to long-term care.

### 2.3. Participants

Among the 12 facilities, 635 nursing home staff were sent a survey questionnaire. Of these, 386 (60.8%) returned the questionnaire.

The sample size was calculated for the primary analysis via the software G*Power 3.1.9.2 [23,24]. Assuming an alpha level of 0.05 and 95% power, the required number of participants to observe an effect size of 0.10 in the psychosocial work environment competence was 277.

### 2.4. Procedure

Each facility was asked to distribute the questionnaires to professional caregivers including nurses, other direct care workers, and care managers, who were asked to evaluate their professionalisms in dementia care and psychosocial work environment. The questionnaires were administered over a 4-week period. Completed questionnaires were returned via mail.

The questionnaires included an introductory section that consisted of a description of the study purpose, an explanation of the voluntary nature of participation, and an assurance of anonymity for the responding professionals and facilities. Unique identification numbers were assigned to the facilities and professionals to preserve anonymity.

The study was approved by the ethics review board at the Tokyo Gakugei University (No. 247, approval on 28 July 2017). All procedures performed in studies involving human participants were in accordance with the ethical standards of the institutional and/or national research committee and with the 1964 Helsinki declaration and its later amendments or comparable ethical standards. Informed consent was not sought from the professionals, as returning the questionnaire implied consent. The institutional review board waived the requirement of informed consent after review of our study protocol because the study involved retrospective reviews.

### 2.5. Measurements

The primary outcome variable was “psychosocial work environment” of the professional caregiver. Psychosocial work environment was assessed using the Social Capital and Ethical Climate at the Workplace (SEW) scale. The SEW scale comprises 20 items that have been developed to evaluate the extent and intensity of associational links or activities, support, reciprocity, and trust within nurses in a Japanese workplace. The scale has three subscales: Social Capital in the Workplace, Ethical Leadership, and Exclusive Workplace Climate. Social capital refers to social relationships that facilitate collective action for mutual benefit [25], and the Social Capital in the Workplace subscale includes 9 items such as “Nurses pursue the collective goals of their workplace”. Ethical Leadership includes 6 items such as “Leaders encourage staff nurses’ ideas in decision making”. Exclusive Workplace Climate includes 5 items such as “Those who disagree with the opinions of those in higher positions of authority become outcasts in the workplace”. Each item is evaluated using a 7-point Likert scale ranging from 1 (totally disagree) to 7 (totally agree). The mean of summed score was calculated per subscale. The higher the mean score for social capital in the workplace and ethical leadership, the greater the participants perceived supportive environment; and the higher the mean score for exclusive workplace climate, the greater the participants perceived negative work environment characteristics. The reliability and validity of the SEW scale have been confirmed [26]. In the present study, Cronbach’s alpha was 0.93 for Social Capital in the Workplace, 0.93 for Ethical Leadership, and 0.83 for Exclusive Workplace Climate.

Variables for quality improvement initiatives included type of home (unit-type or traditional home), presence of an additional benefit for dementia care under the public long-term care insurance program in the facility, and professionalism in dementia care among professional caregivers. Professionalism was measured using the Professionalism subscale of the Japanese version of the Sense of Competence in Dementia Care Staff (SCIDS) scale. The SCIDS scale items are scored using a 4-point Likert scale ranging from 1 = “not at all” to 4 = “very much”. Item scores are summed to form the scale score, with greater scores indicating a higher level of sense of competence [27]. The SCIDS scale contains four subscales that assess professionalism, building relationships, care challenges, and sustaining personhood. The reliability and validity of the Japanese version of the SCIDS scale have been confirmed [28]. In the present study, Cronbach’s alpha for the Professionalism subscale was 0.76.

The covariates were professional characteristics. Professional characteristics included age, gender, education level, profession, and work experience. Profession was categorized into nurse (registered nurse or certified nursing assistant), certified care worker, home helper, and other care worker. In Japan, a license for direct care work with the older adults includes certified care worker and home helper, with certified care worker having a higher qualification. Work experience was defined as the number of years of practice in care for older adults.

### 2.6. Statistical Methods

A correlation coefficient was calculated between professionalism in dementia care and psychosocial work environment subscales.

Between-group difference was examined in professionalism in dementia care and psychosocial work environment subscales according to unit-type/traditional home and presence of dementia care benefit.

A linear regression analysis was conducted using each subscale of psychosocial work environment as the dependent variable, and professionalism in dementia care, type of nursing home, and presence of dementia care benefit as the independent variables. Professional characteristics and location of facility (ratio of active job openings to applicants) were included as covariates. As data were taken from professional caregivers nested by facility, an intraclass correlation coefficient (ICC) was calculated as the proportion of variance of the between-facility variance over the total variance in a null model of multilevel regression. The ICC was 0.046–0.047, so that a clustering between professional caregivers within a facility was considered to be negligible. Thus, multiple regression analysis was adapted to determine the professional and organizational characteristics related to psychosocial work environment. All statistical analyses were conducted using STATA SE for Windows, version 15.1 (StataCorp, College Station, TX, USA). The two-tailed significance level was set at 0.05.

## 3. Results

### 3.1. Participants’ Characteristics

Of the 386 questionnaires returned, 21 were excluded due to incomplete information. The final sample included in the analysis consisted of 365 questionnaires completed by professionals from the 12 facilities. Differences between the characteristics of the included and the excluded questionnaires were determined. A significant difference was observed in the percentage of male (66.3% vs. 42.9%, χ^2^ (1) = 4.80, *p* = 0.028) and certified care workers (65.8% vs. 14.3%, χ^2^ (3) = 82.07, *p* < 0.001).

The professional caregivers comprised 53 (14.5%) nurses, 240 (65.8%) certified care workers, 44 (12.1%) home helpers, and 28 (7.7%) other care workers. They had an average age of 40.2 years (SD = 12.3) and 242 caregivers were male (66.3%). The mean years of work experience was 10.0 years (SD = 8.0). Of the participants, 66 (18.1%) graduated from university or graduate school, 192 (52.6%) from vocational school or college, and 107 (29.3%) from high school or junior high school.

### 3.2. Outcome Data

For all 365 participants, the mean scores were 12.6 (SD = 2.4) for professionalism in dementia care, 44.9 (SD = 9.6) for social capital in the workplace, 29.6 (SD = 7.3) for ethical leadership, and 15.8 (SD = 6.1) for exclusive workplace climate. Correlations with professionalism in dementia care were 0.29 (*p* < 0.001) for social capital in the workplace, 0.26 (*p* < 0.001) for ethical leadership, and −0.18 (*p* < 0.001) for exclusive workplace climate.

There was no significant mean difference between the unit-type homes and traditional homes for professionalism in dementia care (*t* (136.48) = 0.75, *p* = 0.454). Professional caregivers of unit-type homes had a significantly higher social capital in the workplace (*t* (129.25) = 2.35, *p* = 0.020), stronger ethical leadership (*t* (141.75) = 2.39, *p* = 0.018), and lower exclusive workplace climate (*t* (132.12) = 1.98, *p* = 0.0497) compared to traditional homes.

Professional caregivers of nursing homes with dementia care benefit indicated a significantly higher professionalism in dementia care (*t* (250.98) = 2.01, *p* = 0.046) compared to nursing homes without the benefit. There were no significant differences in social capital in the workplace (*t* (282.55) = 0.56, *p* = 0.579), ethical leadership (*t* (254.44) = 0.30, *p* = 0.766), or exclusive workplace climate (*t* (276.70) = 1.29, *p* = 0.198) between nursing homes with dementia care benefit and homes without the benefit.

### 3.3. Main Results

The multiple linear regression analysis revealed that high professionalism in dementia care and unit-type nursing home were significantly associated with higher social capital in the workplace, stronger ethical leadership, and lower exclusive workplace climate. There were no significant associations between presence of dementia care benefit and social capital in the workplace, ethical leadership, or exclusive workplace climate. Higher social capital in the workplace was also observed in professionals of older age, male professionals, nurses, or home helpers compared to certified care workers, professionals who were educated at a vocational school/college rather than junior or high school, and professionals with shorter work experience. Stronger ethical leadership was found in male professionals, nurses or home helpers compared to certified care workers, and professionals with shorter work experience. Lower exclusive workplace climate was observed in male professionals, and professionals with shorter work experience (Table 2).

## 4. Discussion

The professional caregivers reported a supportive psychosocial work environment when they had high professionalism in dementia care. In previous studies, good quality dementia care has been reported to be associated with psychosocial work environment [11,12,13]. Because professional caregivers were sometimes unsure about the outcome of their caregiving to persons with dementia, mutual relationships with other professionals are essential to understand the person with dementia and monitor the quality of care [28]. Our study further implies that enhanced professionalism in dementia care may have had a positive impact on the psychosocial work environment.

The professional caregivers reported a positive psychosocial work environment when they worked in unit-type homes rather than traditional homes. Previous studies have shown that unit-type homes improved the ability to treat responsive behavior [19] and enhanced quality of life of residents with dementia [21]. Since professionals in unit-type homes are assigned to a team corresponding to each unit of residents, unit-type homes usually have team leaders to supervise other professionals in the team. The team-based structure may have contributed to constructing social capital and ethical leadership and to reducing exclusive workplace climate. The national government has recommended all traditional homes be renovated as unit-type homes. However, more than half of all nursing homes are traditional homes [16]. As renovation of traditional homes will be at a high cost, the benefit schedule and copayment are higher in unit-type homes rather than traditional homes. Thus, older adults and families preferred traditional homes, resulting in a longer waiting list for admission compared to unit-type homes [19,20]. These factors would have inhibited provider competition and motivation to introduce small-scale environment into their nursing homes. Financial support for renovation of traditional homes should be incorporated in quality improvement initiatives for further promotion of unit-type homes.

Presence of additional benefit for specialized dementia care did not significantly correlate with psychosocial work environments, although professionalism in dementia care was higher among nursing homes with the dementia care benefit. Some facilities may have had the aim of increasing their revenues with the additional benefit, as opposed to improving the quality of dementia care. Successful quality improvement in dementia care rested on the active engagement of caregivers and the continuing provision of tailored interventions and support [29,30,31,32]. The implementation of a quality improvement program should be incorporated into the additional benefit for dementia care.

Clustering of professional caregivers within a facility was small, suggesting that perception of psychosocial work environment differed between professionals even in the same facility. Nurses, home helpers, and professionals with shorter length of work experience were related to high social capital and strong ethical leadership. Male professionals also reported a better psychosocial work environment compared to female professionals. The majority of care staff in special nursing homes are females and certified care workers [16]. Nurses in special nursing homes usually played a role in managing certified care workers who are not allowed to provide medical intervention to nursing home residents. Perceived psychosocial work environment differs according to sex [33] and position (manager or employee) in the organization [34]. Organizational strategies to foster supportive psychosocial work environment should pay specific attention to females, certified care workers, and professionals with longer work experience in special nursing homes.

The results of our study have certain limitations. The sample may have a bias with regard to those who chose to complete the questionnaire and return it. Additionally, participating facilities were relatively open-minded organizations with positive psychosocial work environment. Therefore, the association between quality improvement initiatives and psychosocial work environment may have been underestimated. The cross-sectional design only provided information about professionals at the time of assessment, limiting any inferences regarding causality. Our final model had a low adjusted R square value, suggesting the presence of other unexplained factors contributing to psychosocial work environment.

## 5. Conclusions

Professional and organizational characteristics regarding quality improvement initiatives in dementia care were associated with supportive psychosocial work environment among professional caregivers of special nursing homes. Supportive psychosocial work environment was also found in unit-type homes, regardless of the presence of additional care benefit for dementia care. Quality improvement initiatives to foster supportive psychosocial work environment should focus on enhancement of professionalism in dementia care with unit-based team building of professional caregivers in special nursing homes. Quality improvement initiatives also should be incorporated into the additional benefit for dementia care.

## Figures and Tables

**Table 1:** The ratio of active job openings to applicants in long-term care in March 2016.

Prefecture	Ratio ^1^	Group
Tottori	1.60	Low
Shiga	2.37	Middle
Osaka	3.56	High
(Reference) national	2.73	--

^1^ Data source: Employment Security Bureau, Ministry of Health, Labour and Welfare.

**Table 2:** Linear regression analysis of psychosocial work environment in 365 professional caregivers of special nursing homes.

Variable	Social Capital		Ethical Leadership		Exclusive Climate	
	Coefficient (95&CI)	*p*-Value	Coefficient (95&CI)	*p*-Value	Coefficient (95&CI)	*p*-Value
Constant	17.29 (9.71, 24.87)	<0.001	13.74 (7.83, 19.64)	<0.001	25.50 (20.38, 30.62)	<0.001
Professionalism in dementia care	1.12 (0.75, 1.50)	<0.001	0.81 (0.51, 1.10)	<0.001	−0.43 (−0.69, −0.18)	0.001
Type of nursing home, unit-type	3.43 (1.02, 5.85)	0.006	3.12 (1.24, 5.00)	0.001	−2.65 (−4.28, −1.02)	0.002
Additional benefit for dementia care	−0.48 (−2.97, 2.01)	0.704	−1.06 (−3.00, 0.87)	0.280	0.69 (−0.99, 2.37)	0.422
Job opening, reference = low						
Middle	1.33 (−3.48, 6.14)	0.587	3.11 (−0.63, 6.85)	0.103	−1.44 (−4.68, 1.81)	0.385
High	3.28 (−1.57, 8.12)	0.184	3.08 (−0.69, 6.85)	0.109	−0.10 (−3.37, 3.17)	0.951
Professional characteristics						
Age, year	0.15 (0.05, 0.25)	0.004	−0.01 (−0.09, 0.07)	0.746	0.002 (−0.07, 0.07)	0.944
Sex, male	2.59 (0.42, 4.75)	0.019	1.77 (0.08, 3.45)	0.040	−1.62 (−3.08, −0.16)	0.029
Profession (reference = certified care worker)						
Nurse	3.75 (0.25, 7.25)	0.036	3.42 (0.70, 6.15)	0.014	−0.91 (−3.27, 1.46)	0.450
Home helper	3.75 (0.75, 6.79)	0.015	2.62 (0.26, 4.97)	0.029	−1.85 (−3.89, 0.19)	0.075
Other care worker	0.32 (−3.29, 3.92)	0.863	0.63 (−2.17, 3.44)	0.657	−0.29 (−2.72, 2.15)	0.816
Education (reference = junior or high school)						
Vocational school/college	3.75 (1.39, 6.11)	0.002	1.77 (−0.07, 3.61)	0.059	−1.56 (−3.16, 0.03)	0.054
University or graduate school	2.04 (−0.88, 4.95)	0.171	1.10 (−1.17, 3.37)	0.343	−1.38 (−3.35, 0.59)	0.168
Work experience, year	−0.25 (−0.43, −0.08)	0.005	−0.20 (−0.34, −0.06)	0.005	0.06 (−0.06, 0.18)	0.332
Adjusted R-square	0.181		0.141		0.070	
F (13, 351)	7.18	<0.001	5.59	<0.001	3.09	<0.001

CI, confidence interval.
